# Pharmacokinetics and transcriptional effects of the anti-salmon lice drug emamectin benzoate in Atlantic salmon (*Salmo salar *L.)

**DOI:** 10.1186/1471-2210-8-16

**Published:** 2008-09-11

**Authors:** Pål A Olsvik, Kai K Lie, Eva Mykkeltvedt, Ole B Samuelsen, Kjell Petersen, Anne-Kristin Stavrum, Bjørn T Lunestad

**Affiliations:** 1 National Institute of Nutrition and Seafood Research, P.O. Box 2029 Nordnes, N-5817 Bergen, Norway; 2 Institute of Marine Research, Fish Disease Group, P.O. Box 1870 Nordnes, 5817 Bergen, Norway; 3 Computational Biology Unit, Bergen Center for Computational Science, University of Bergen, Thormøhlensgt 55, N-5008 Bergen, Norway; 4 Department of Clinical Medicine, University of Bergen, N-5021 Bergen, Norway

## Abstract

**Background:**

Emamectin benzoate (EB) is a dominating pharmaceutical drug used for the treatment and control of infections by sea lice (*Lepeophtheirus salmonis*) on Atlantic salmon (*Salmo salar *L). Fish with an initial mean weight of 132 g were experimentally medicated by a standard seven-day EB treatment, and the concentrations of drug in liver, muscle and skin were examined. To investigate how EB affects Atlantic salmon transcription in liver, tissues were assessed by microarray and qPCR at 7, 14 and 35 days after the initiation of medication.

**Results:**

The pharmacokinetic examination revealed highest EB concentrations in all three tissues at day 14, seven days after the end of the medication period. Only modest effects were seen on the transcriptional levels in liver, with small fold-change alterations in transcription throughout the experimental period. Gene set enrichment analysis (GSEA) indicated that EB treatment induced oxidative stress at day 7 and inflammation at day 14. The qPCR examinations showed that medication by EB significantly increased the transcription of both HSP70 and glutathione-S-transferase (GST) in liver during a period of 35 days, compared to un-treated fish, possibly via activation of enzymes involved in phase II conjugation of metabolism in the liver.

**Conclusion:**

This study has shown that a standard seven-day EB treatment has only a modest effect on the transcription of genes in liver of Atlantic salmon. Based on GSEA, the medication seems to have produced a temporary oxidative stress response that might have affected protein stability and folding, followed by a secondary inflammatory response.

## Background

One of the major problems in aquaculture of salmonids such as Atlantic salmon (*Salmo salar L*.) and rainbow trout (*Oncorhynchus mykiss*) is production loss due to ectoparasites like sea lice [1], which are easily spread between individuals in densely populated sea cages. The term sea lice is collectively used for ectoparasitic copepods (Copepoda, Caligidae) found on marine fish species, including salmonid fish. The main species of concern in North Atlantic marine salmonid aquaculture causing infections are *Lepeophtheirus salmonis *and *Caligus elongatus*. The parasites undergo several developmental stages, including planktonic stages and stages where the parasite is attached to or moving on the fish surface, feeding on mucus and blood [2,3]. The main effects of sea lice infestations are general stress and osmoregulatory problems due to disruption of the skin by the feeding behaviour of the parasites [4].

Emamectin benzoate (EB) is currently the dominant peroral pharmaceutical drug used for the treatment and control of sea lice infestations on salmonids. EB is commonly used due to its effectiveness against all stages of sea lice infection [5]. EB is the active ingredient in SLICE, a commercial drug commonly used for sea lice control in Atlantic salmon farming. It is commonly used in many countries including Norway, UK, Canada and Chile that are producing large quantities of Atlantic salmon in aquaculture [6]. EB (4''-deoxy-4'' epi-methylamino-avermectin B_1_) is a semi-synthetic avermectin, a group of insecticides that were originally isolated from soil microorganisms [7] and used for the control of insect pests in edible crops [8]. The mechanism of action of the avermectins in invertebrates is the binding to glutamate-gated chloride channels leading to an influx of chloride ions, thus giving a hyperpolarized cell. An additional mechanism is increasing the production of the inhibitory neuro-transmitter GABA at nerve endings, which prolongs the binding of GABA to the receptor, thus mediating the same effect. In invertebrates, avermectins act on muscle cells and synapses in the peripheral nervous system, causing paralysis and eventually death of the parasite. In mammals however, the toxic effect is low since the avermectins do not cross the mammalian blood brain barrier, and thus do not affect GABA-mediated neurons at in the central nervous system (CNS). According to the EU legislation described in the directive EC 2377/90, EB thus has been given a Maximum Residue Limit (MRL) in edible tissue of 100 ng/g. In fish, the blood brain barrier is not as impermeable as in mammals and CNS depression and deaths have been reported in salmon using avermectin at therapeutic doses.

Orally administered EB is readily absorbed and distributed to tissues in salmonids [9]. Metabolism of EB in fish is rather limited, resulting in sustained tissue concentrations. Eventually, absorbed and metabolized EB is excreted in feces via bile in the liver, a process that probably involves enterohepatic recirculation of EB, as observed in SLICE-treated rainbow trout [9]. Possible effects of EB medication on the fish is therefore most likely to be manifested in hepatocyte cells in the liver, although very little is known about toxicological effects of EB on salmonid fishes. Roy et al. [10] examined the tolerance of Atlantic salmon and rainbow trout to EB exposure, and signs of EB toxicity included lethargy, dark coloration, inappetance and loss of coordination but no pathognomonic signs of toxicity during gross necropsy or histopathological examinations [5]. With the rapidly increasing number of known gene sequences in many species, transcriptional analysis has become one of the cornerstones of modern biology. So far more than 430 000 Atlantic salmon gene sequences from Atlantic salmon have been deposited in the Genbank, making it possible to search for biomarkers based on transcriptional responses to external stimuli with techniques like qPCR and microarray. The Consortium for Genomic Research on All Salmon Project (cGRASP) [10]. at the University of Victoria, Canada, has produced a large-scale cDNA microarray containing about 16000 clones that can be used in search of genome-wide responses to environmental stressors and medication in salmonid fishes [11].

The aim of this work was to examine to which degree EB has a toxic effect or is imposing stress on Atlantic salmon treated with the anti-salmon lice medication SLICE that contains EB as the active component. Juvenile Atlantic salmon were orally administered a daily EB dose of 50 μg/kg fish, mixed into the feed over a standard seven day medication period [12]. Tissue residues of EB were measured in liver, muscle and skin at day 7 (end of the treatment period), 14 and 35. Microarray and qPCR techniques were used to search for transcriptional biomarkers for EB toxicity or stress induction in liver of medicated fish.

## Methods

### Experimental fish

**Figure 1:**
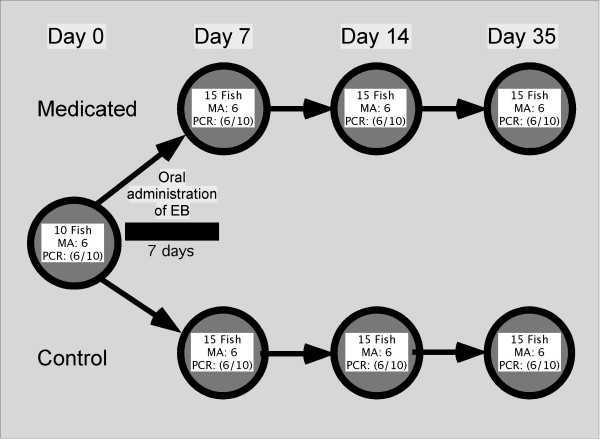
Experimental design. Treatment, sampling and number of fish used for gene transcription analysis. Total number of individuals sampled, number of samples used for microarray analysis (MA) and qPCR (PCR) are given for each sampling time. A total of 100 Atlantic salmon were used in the experiment. RNA was extracted from 70 individuals, 10 from each of the 7 groups. For microarray (MA, n = 6) and qPCR verification analysis the same 6 individuals were used; a total of 42 samples were analyzed, with 6 biological replicates from each group (3 males and 3 females). For array-independent qPCR analysis of HSP70 and GST, n = 10 in each group (total n = 70). EB = Emamectin benzoate.

Juvenile Atlantic salmon (*Salmo salar L*.) were obtained from the Institute of Marine Research, Bergen, Norway and maintained in flow-through seawater tanks (32‰ salinity, mean temperature 9.1°C). Prior to challenge, fish were randomly assigned to two 500 L tanks and raised in these throughout the experiment. One group of fish was kept as a control, whereas the other was treated with EB. An overview of the experimental design can be seen in Fig. 1.

### Fish treatment

The medicated feed was produced by adding EB in a concentration of 1 mg/100 g to dry feed. The experimental fish were administered an oral standard medication regime of 50 μg EB per kg fish daily for seven days. The feeding rate was 0.5% body weight per day. Ten individual fish were collected at time 0 (untreated), thereafter 30 individual fish were collected at day 7, 14 and 35, from one control (n = 15) and one EB treated group (n = 15) at each time. A total of 100 fish were used in the experiment. No mortality was observed throughout the experiment. On average, the fish weighed 132 ± 21 g at time 0, 147 ± 25 g at day 7, 147 ± 27 g at day 14 and 189 ± 28 g at day 35 (mean ± SD). Non-medicated fish were fed a commercial dry pellet salmon feed at 0.5% body weight per day. The fish were killed by a blow to the head, after being removed from the tanks. No anesthetics were used.

### Tissue sampling

Fish tissues for EB analysis were dissected out and stored at -20°C before further processing. Approximately 100 mg of tissue was sliced off the liver and immediately flash-frozen in liquefied nitrogen for RNA extraction. Tissue specimens for RNA extraction were stored at -80°C before further processing.

### Emamectin benzoate analysis

Fish tissue samples (1.5 g) were homogenized in 1 ml of 0.9% NaCl in water and 300 μl of a solution of 1.5 μg/ml of ivermectin (internal standard) in methanol. After homogenization, 6 ml acetonitrile was added followed by shaking, sonication for 10 min and centrifugation. To the supernatant, 5 ml n-heptane was added followed by thorough shaking and removal of the n-heptane layer. Water (20 ml) was then added and the sample was subjected to solid phase extraction using a OASIS HLB 6 cc cartridge (Waters Corporation, Milford, Mass. USA) conditioned with 5 ml methanol followed by 4 ml of distilled water. After loading the sample, the column was washed with 3 ml of 33% acetonitrile in water and 3 ml of n-heptane prior to elution of EB by 3 ml of methanol. The column was dried by vacuum before the final elution of EB. The eluate was evaporated to dryness under a stream of nitrogen using a Reacti-Therm heating unit at 50°C and a Reacti-Evaporating unit (Pierce, Rockford, IL, USA). The dry residue was reconstituted in 300 μl methanol and 100 μl n-heptane, shaken for 30 sec and centrifuged for 3 min at 2000 rpm (Eppendorf 5810 R, Hamburg, Germany). The methanol fraction was filtered through a 0.45 μm syringe filter prior to injection into the high performance liquid chromatographic system (HPLC). Ivermectin and EB were supplied by Sigma-Aldrich Chemie (Steinheim, Germany). Methanol, acetonitrile, ammonium-acetate (all HPLC-grade) and n-heptane, acetic acid (100%; PA-grade) were all from Merck (Darmstadt, Germany). The water used was purified with a MilliQ water purification system (Millipore, Bedford, MA, USA). Standards were made by dissolving 50 mg ivermectin and emamectin in 10 ml of methanol.

The concentration of EB in muscle was determined using an Agilent 1100 series HPLC connected to a MSD Quadropole mass-spectrometer (Agilent Technologies, Waldbronn, Germany). The analytical column was a 125 × 4 mm LiChrosphere C-18, 5 μm with a 4 × 4 mm LiChrosphere 100 RP-18, 5 μm guard column (Agilent Technologies, Palo Alto, CA, USA). The column temperature was 35°C. The mobile phase contained 50 mM ammonium acetate: acetonitrile: MilliQ water (5:75:20) and the elution profile was isocratic. The flow rate was 0.8 ml/min, giving elution times of 9.2 min for EB and 12.1 min for ivermectin. The injected sample volume was 40 μl. The mass-spectrometer was tuned in positive selected ion monitoring (SIM) mode with ion peaks at *m/z *of 982.5 for ivermectin and 886.4 for emamectin. The following tune parameters were used: APCI vaporizer temperature 450°C, corona current 5.0 μA, capillary voltage 2500 V, nebulizer pressure 60 psi, drying gas 4 l/min and fragmentor voltage 70 V. The calibration curve for emamectin was prepared in replicate by spiking muscle samples with standard solutions of emamectin to yield 2.5, 12.5, 25.0 and 50.0 ng/g. Ivermectin (300 ng/g) was added to each sample and acted as internal standard. The limit of quantification (LOQ) method was determined to be 5.0 ng/g and the limit of detection (LOD) to be 2.5 ng/g. The method for detection and quantification of emamectin used in this study are accredited in accordance with the international standard ISO/IEC 17025:1999 [13].

### RNA extraction

Liver tissues from 70 individuals were thoroughly homogenized before RNA extraction with zirconium beads (4 mm) in a MM 301 homogenizer (Retsch GmbH, Haan, Germany). Total RNA was extracted using Trizol reagent (Invitrogen, Life Technologies, Carlsbad, CA, USA), according to the manufacturer's instructions and stored in 50 μl RNase-free MilliQ H_2_O. Genomic DNA was eliminated from the samples by DNase treatment using DNA-free according to the manufacturer's description (Ambion, Austin, TX, USA). The RNA was then stored at -80°C before further processing. The quality of the RNA was assessed with the NanoDrop^® ^ND-1000 UV-Vis Spectrophotometer (NanoDrop Technologies, Wilmington, DE, USA) and the Agilent 2100 Bioanalyzer (RNA 6000 Nano LabChip^® ^kit, Agilent Technologies, Palo Alto, CA, USA).

### Microarray analysis

A total of 42 RNA samples out of the total of 70 samples were prepared for microarray analysis. The RIN values for these 42 samples ranged from 9.3 to 10.0 (mean ± SD: 9.9 ± 0.2). RNA was extracted from six fish, three males and three females, from each group as shown in Fig. 1. cDNA synthesis was made with the 3DNA Array 350 HS kit according to the manual (Genisphere Inc., Sterling Drive, PA, USA). A common reference design was utilized with the experimental samples in the Cy5 channel and the reference samples in the Cy3 channel, hybridized to the cGRASP v.2.0 16 K cDNA microarray [11]. Every 42 samples, both experimental and control were individually hybridized with the common reference, with 6 biological replicates for each group. No technical replicates were run. Hybridization was performed with a HS 4800 TM Hybridization station (Tecan, Männedorf, Switzerland), whereas scanning was performed with a LS Reloaded Scanner (Tecan, Männedorf, Switzerland). GenePix software (Axon Instruments, Union City, CA, USA) was used to analyze the scans. The reference was prepared from a pool of liver RNA from 50 individuals collected in an independent Atlantic salmon experiment.

The data files from GenePix v.5.1 were processed using J-Express Pro v.2.8 [14] to filter and normalize the hybridization data and compile the transcription matrix (gene by sample) for further analysis. The foreground signal intensity values for each channel were extracted for each spot from the data files according to the GenePix software manual (Axon Instruments, Union City, CA, USA). All flagged and control spots were filtered out before the data were normalized using global lowess [15]. After normalization, weak spots with FG < BG + 1.5*BG_SD in both channels were filtered out. All arrays were then compiled into a single expression profile data matrix containing the normalized log ratios of the two foreground signal intensities. Rows with more than 75% missing values were removed from the matrix, and the remaining missing values were estimated using LSimpute_adaptive [16]. Finally the data was divided into sub-datasets for the individual sampling days, and genes with at most 25% estimated missing values were allowed in the final expression matrix.

The search for differentially expressed genes was performed both on a single gene and gene set level. A two class paired SAM [17] v.2.1 analysis, as implemented in J-Express, was used to look for differentially expressed genes on a gene by gene basis, while GSEA [18,19] also implemented in J-Express, was used to look for sets of genes sharing common characteristics that were differentially expressed between the classes examined. Gene sets were created on the basis of Gene Ontology (Gene Ontology Consortium [20]), by mapping the GO annotations in the cGRASP v.2.0 annotation file (dated August 2007) to the GO accession numbers in the Gene Ontology OBO file dated 08.06.07. *Parameters of GSEA *– Parallel analyses were run with probes collapsed to genes, using the gene description column, as well as non-collapsed expression matrix as input. Gene sets smaller than 10 and larger than 500 were excluded from the analysis. GSEA was run with SAM score as the ranking statistic. Significance of the gene set analysis was tested by permuting the scores over the genes (10000 iterations).

### qPCR

In order to verify the microarray data, qPCR was used to quantify the transcriptional levels of eight differentially regulated genes in the same 42 RNA samples as used for microarray analysis. For verification, genes were picked from the statistical analysis of microarray (SAM) lists; two up-regulated and one down-regulated genes based on q-statistics [GenBank:CB511007;CB509633;CB514814], and one up-regulated and three down-regulated genes based on fold-change [GenBank:CA056074;CB488966;CA053315;CB491960]. In addition, one gene was picked that appeared to be un-regulated but was included in several gene sets (HLA class II histocompatibility antigen, gamma chain [GenBank:CK990815]. qPCR assays were designed using Primer Express 2.0 software (Applied Biosystems, Foster City, CA, USA) to select appropriate primer sequences from gene sequences included on the array. Since some of the primers did not span exon-exon borders, all RNA samples were subjected to DNase treatment to avoid genomic DNA contamination. PCR primer sequences, GenBank accession numbers and amplicon lengths of the genes selected for qPCR verification are shown in Table 1.

**Table 1:** PCR primers and TaqMan MGB probes, Genbank accession numbers, amplicon sizes and fold changes (microarray and qPCR verification) for the studied genes.

**Gene**	**Accession no.**	**Forward primer (5'-3')**	**Reverse primer (5'-3')**	**TaqMan MGB probe**	**Amplicon size (bp)**	**Microarray**	**qPCR**
Thrombospondin 4 precursor Day 14	CB509633	GCAGCGGTACTTTAGGTTGGA	ATCAGGGCCCGTTTCTATGA		141	-1.6	-1.7
Purine nucleoside phosphorylase Day 14*	CB514814	GCCCCCTTCATGGGTACAC	AACGCCTGAACGAACGAATG		135	2.1	1.6
CYP2B19 Day 7	CA053315	AGCCTGTGACCTCTCCACAGTAA	CGGCACAAAACCTCCAGAAG		134	-3.2	-2.2
CYP2A5 Day 7	CB491960	AGGTTTGGTGCCGGTGAAA	ATGATGGATTCTTTGCTTTTGGA		125	-3.2	-1.7
Low affinity immunoglobulin epsilon Fc receptor Day 14*	CB511007	CCACTCACAGGGCACATCAA	GTGGTCAGATGGGTCCAGATTT		133	2.8	2.1
NADH-ubiquinone oxireductase chain 1 Day 7	CB488966	GGCCGGCACGAGTAGTCA	GGCAGTGGCACAAACCATTT		133	-3.3	-1.2
Sequestosome-1 Day 14*	CA056074	GGGACAGAAAGAGAAGGCAGTATT	GCCCTGGACACCATCCACTA		131	1.3	1.1
HLA class II histocompatibility antigen, gamma chain Day 14*	CK990815	TTATATGCTGTCCGAAGGCAAA	CCCTCCCCCAAAAAATACACA		141	1.1	1.2
Heat shock protein 70	AJ632154	TCAACGATCAGGTCGTGCAA	CGTCGCTGACCACCTTGAA	CCGACATGAAGCACTGG	141		
Glutathione S-transferase pi	BQ036247	ATTTTGGGACGGGCTGACA	CCTGGTGCTCTGCTCCAGTT	TTCTCGACAAAGCTC	81		
β-actin	BG933897	CCAAAGCCAACAGGGAGAAG	AGGGACAACACTGCCTGGAT	TGACCCAGATCATGTTT	91		
EF1AB	BG933853	TGCCCCTCCAGGATGTCTAC	CACGGCCCACAGGTACTG	CCAATACCGCCGATTTT	59		
ARP	AY255630	TCATCCAATTGCTGGATGACTATC	CTTCCCACGCAAGGACAGA	CAAATGTTTCATTGTCGGCG	101		

* = up-regulated genes

A two-step qPCR protocol was developed to measure the mRNA levels of the eight target genes and the three reference genes (β-actin, elongation factor 1A_B _(EF1A_B_) and acidic ribosomal protein (ARP)) in liver tissue of Atlantic salmon. The reverse transcription reactions were run in duplicate on 96-well reaction plates with the GeneAmp PCR 9700 machine (Applied Biosystems) using TaqMan Reverse Transcription Reagent containing Multiscribe Reverse Transcriptase (50 U/μl) (Applied Biosystems). Twofold serial dilutions of total RNA were made for efficiency calculations. Five serial dilutions (1000 – 63 ng) in triplicates were analyzed by qPCR in separate sample wells and the resulting crossing thresholds (Cts) recorded. Total RNA input was 500 ng in each reaction for all genes. No template control (ntc) and RT-control (a duplicate RNA sample analysis where only the RT enzyme is left out) reactions were run for quality assessment. RT-controls were not performed for every individual sample, but were run for each assay or gene, with the same sample as used to make the dilution curves on the 96 well plates. Reverse transcription was performed at 48°C for 60 min by using oligo dT primers (2.5 μM) for all genes in 30 μl total volume. The final concentration of the other chemicals in each RT reaction was: MgCl_2 _(5.5 mM), dNTP (500 mM of each), TaqMan RT buffer (1×), RNase inhibitor (0.4 U/μl) and Multiscribe reverse transcriptase (1.67 U/μl).

2.0 μl cDNA from each RT reaction for all genes was transferred to a new 96-well reaction plate and the qPCR run in 20 μl reactions on the LightCycler^® ^480 Real-Time PCR System (Roche Applied Sciences, Basel, Switzerland). qPCR was performed by using SYBR Green Master Mix (LightCycler 480 SYBR Green master mix kit; Roche Applied Sciences), which contains FastStart DNA polymerase and gene-specific primers (500 nM each). PCR was achieved with initial denaturation and enzyme activation for 5 min at 95°C, followed by 40 cycles of 10 s denaturation at 95°C, 20 s annealing at 60°C and 30 s elongation at 72°C. The *geNorm *VBA applet for Microsoft Excel was used to determine a normalization factor from the three examined reference genes used to calculate mean normalized expression (MNE) for ACTB, EF1A_B _and ARP. The Ct values were transformed to quantities using standard curves, according to the *geNorm *manual [21]. *geNorm *determines the individual stability of a gene within a pool of genes, and calculates the stability according to the similarity of their transcription profile by pair-wise comparison, using the geometric mean as a normalizing factor. The gene with the highest M, i.e. the least stable gene, is then excluded in a stepwise fashion until the most stable genes are determined. Here a normalizing factor based on all three examined reference genes was used to calculate the MNE.

Independent and a priori of the microarray analysis, the transcriptional levels of two additional genes that were assumed to be affected by the EB treatment were quantified by qPCR. In this examination, mRNA levels were quantified in 10 individuals from each group, as opposed to n = 6 in the microarray and subsequent qPCR verification analysis. The PCR primers and probes used to quantify these transcripts were based on Genbank sequences not included on the cGRASP array. PCR primer sequences used for quantification of the genes encoding β-actin, EF1A_B _and ARP, used as reference genes, and HSP70 and GST π, were based on the following Genbank accession numbers [GenBank:BG933897;BG933853;AY255630;AJ632154;BQ036247], respectively. TaqMan 3'-Minor groove binder-DNA (MGB) probes were used to quantify these genes as described by Olsvik et al. [22]. Analyzed with *geNorm *[21], EF1A_B _was found to be the most stable reference gene, and therefore used to calculate mean normalized expression with the *qGene *tool [23]. Except for the β-actin and EF1A_B _assays, the PCR primers or probes did not span exon-exon borders. All RNA samples were therefore treated with DNase (Ambion) according to the manufacturers instructions. Amplified PCR products were sequenced as described above and subsequently compared to the database using BLAST to ensure that the correct mRNAs were analyzed.

### Statistics

The GraphPad Prism 4.0 (GraphPad Software, Inc., San Diego, CA, USA) and J-Express Pro (Molmine, Bergen, Norway) software were used for the statistical analyses in this work. Linear regression and correlation analysis were performed with GraphPad Prism, whereas significance of microarray (SAM) and gene set enrichment analysis (GSEA) were performed with J-Express Pro. A wide range of statistics can be used to calculate differential transcription between two groups. T-test and S-score (regularized t-score) that is used in SAM [17] are commonly used tests. SAM was used here because it is well adapted to microarrays and reports q-values for assessment of statistical significance of the results after correcting for multiple testing. Gene set enrichment analysis (GSEA) calculates an enrichment score (ES) for a given gene set using rank of genes and infers statistical significance of each ES against ES background distribution calculated by permutation of the original data set [18,24]. For qPCR validation an alpha level of 0.05 was considered significant, while for SAM and GSEA a threshold of 10% false discovery rate was used to control for multiple testing in the genome wide data analysis.

## Results

### Pharmacokinetics

**Figure 2:**
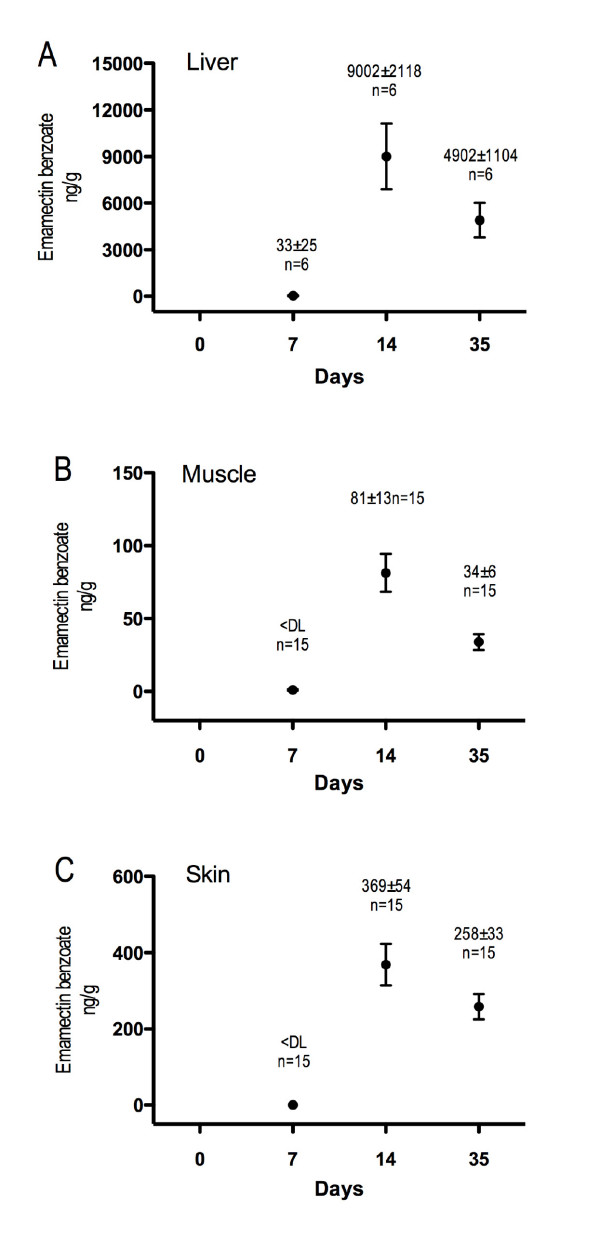
Pharmacokinetics. Uptake and drug residues of EB in Atlantic salmon, (*Salmo salar L*.), administered a standard daily oral dose of 50 μg/kg for 7 days (mean ± SEM). The levels of EB were quantified in 6 individuals in liver (the same individuals as used for microarray analysis) and 15 individuals in muscle and skin tissues. <DL: below detection limit.

The concentrations of EB in liver, muscle and skin at days 7, 14 and 35 after the initiation of emamectin benzoate medication are shown in Fig. 2. At day 7 the mean concentration of EB in samples of liver was 33 ng/g, whereas the mean concentrations in muscle were 1 ng/g. The skin did not contain EB in concentrations above the level of detection (LOD) at day 7. At day 14 the mean concentrations in liver, muscle and skin were 9002, 81 and 369 ng/g, respectively. The corresponding mean concentrations at day 35 were 4902, 34 and 258 ng/g respectively. Overall, the EB concentrations were highest at day 14, and had dropped by day 35.

### Gene expression profiling data

**Figure 3:**
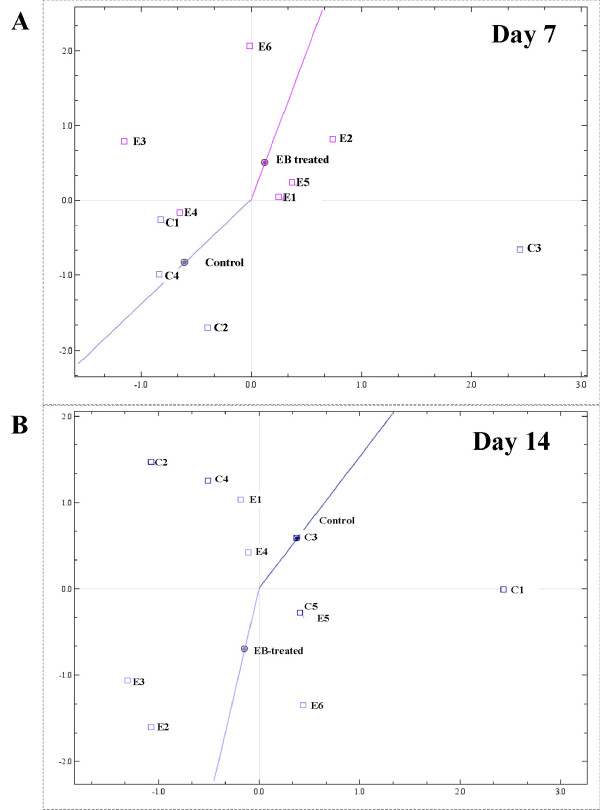
Correspondence analysis (CA) of arrays after day 7 (A) and day 14 (B). C = controls, E = EB-treated. n = 6, except in the control group at day 7 (n = 4) and the control group at day 14 (n = 5).

The microarray data revealed only small alterations in transcript levels in the EB-medicated fish compared to the control at day 7, 14 and 35. The SAM analysis showed that only three genes were significantly differentially expressed at day 7; type IV antifreeze protein precursor, MHC class I antigen alpha chain BL3-7 and an unknown gene. At day 14 only two genes displayed differential transcription levels, thrombospondin-4 precursor and purine nucleoside phosphorylase. Correspondence analysis (CA) is used to look for the greatest co-variances (between samples and genes) in the data. The plot gives a global view of the data and can reveal clustering of samples according to their biological groups. At day 7, the CA plot showed that there is a tendency of separation of the EB-medicated and the control groups (Fig. 3A). Since the pharmacokinetic measurements reveal the highest EB concentration in the liver at day 14, we expected to see more differences between the two groups at this time point. Although it was not possible to draw a straight line between the points to separate the two groups, the CA plot clearly showed a tendency of separation between the two groups at day 14 (Fig. 3B). On the contrary, there did not seem to be major differences between the two groups on day 35 based on the global CA plot. The microarray data has been submitted to ArrayExpress with MIAME required documentation and is available under the accession number E-BASE-9.

### Gene set enrichment analysis

The overall effects of EB treatment on hepatic transcriptional levels were modest, which may have reduced the significance of biologically relevant genes because their signal intensities were relatively low. It was therefore decided to evaluate the data using the newest Gene Ontology annotations of the arrayed probes to identify significant biological changes. Table 2 shows gene sets up-regulated at day 7, ordered by the normalized enrichment score, with a false discovery rate (FDR) cutoff of approximately 10%, meaning that we expected a maximum of 1 out of 10 gene sets to be false positives. Probes annotated to the same gene were not collapsed before performing GSEA that resulted in the FDR values shown in Table 2. Multiple probes on the array mapped to the same gene in a gene set will produce more significant FDR values, but as long as the Atlantic salmon genome is relatively poorly annotated, i.e. with unknown gene function, isoforms and splice variants, this strategy was selected in order not to lose valuable biological information. By collapsing data at the level of gene symbols, one risks an unintended merging of information from every pair of paralogue genes in the salmon genome. On the other hand, by not collapsing the data, one risks the pitfall of some gene sets being favored in the statistical calculation due to several probes for the same gene contributing to a particular gene set. A similar biological picture of inflammation and oxidative stress was also seen in the collapsed data set, but not at the same 10% FDR level of significance. Up-regulated gene sets at day 14 are shown in Table 3. No gene sets were significantly down-regulated at day 7 or at day 14. Table 4 shows up- and down-regulated gene sets at day 35, again with a FDR cutoff of approximately 10%.

**Table 2:** Gene sets significantly up-regulated after 7 days of medication (day 7).

**Rank**	**Gene Sets – Up-regulated**	**Size**	**Nom P-value**	**FDR (%)**
1	Protein disulfide isomerase activity	26	0.0	1.72
2	Intramolecular oxidoreductase activity, transposing S-S bonds	26	0.0	0.86
3	Peptidyl-asparagine modification	16	0.0	3.2
4	Protein amino acid N-linked glycosylation via asparagine	16	0.0	2.4
5	Intramolecular oxidoreductase activity	36	0.0	2.89
6	Protein localization	37	0.0	11.7
7	Isomerase activity	52	0.0	10.9
8	Peptidyl-amino acid modification	23	0.0	10.79
9	Oligosaccharyl transferase complex	19	0.0	10.66

Gene set enrichment analysis (GSEA) with a false discovery rate (FDR) cut-off of approximately 10%. Nominal P values are also shown, as well as gene set sizes (number of genes in each gene set). No gene sets were significantly down-regulated after 7 days of medication, with a FDR lower than about 10%.

**Table 3:** Gene sets significantly up-regulated 7 days after the end of the medication period (day 14).

**Rank**	**Gene Sets – Up-regulated**	**Size**	**Nom P-value**	**FDR (%)**
1	Prostanoid biosynthetic process	19	0.0	0.38
2	Prostaglandin biosynthetic process	19	0.0	0.19
3	Cytokine binding	21	0.0	0.37
4	Regulation of macrophage activation	15	0.0	0.44
5	Ecosanoid biosynthetic process	29	0.0	0.49
6	Ecosanoid metabolic process	29	0.0	0.41
7	Carbohydrate binding	34	0.0	0.47
8	Macrophage activation	18	0.0	0.6
9	Myeloid leukocyte activation	18	0.0	0.54
10	Sugar binding	29	0.0	0.98
11	Monosaccharide binding	16	0.0	1.79
12	Glutamate dehydrogenase [NAD(P)+] activity	14	0.0	2.99
13	Leukocyte activation	37	0.0	3.58
14	Activation of MAPK activity	12	0.0	4.6
15	Intracellular protein transport	41	0.0	5.8
16	Protein amino acid N-linked glycosylation via asparagines	17	0.0	5.94
17	Peptidyl-asparagine modification	17	0.0	5.59
18	Oxidoreductase activity, acting on the CH-NH2 group of donors, NAD or NADP as acceptor	17	0.0	5.54
19	Mannose binding	12	0.01	5.34
20	Antigen processing and presentation of exogenous peptide antigen via MHC class II	24	0.0	8.97
21	Antigen processing and presentation of peptide antigen via MHC class II	24	0.0	8.55
22	Transition metal ion binding	31	0.0	8.69
23	Peptidyl-amino acid modification	24	0.01	10.94

Gene set enrichment analysis (GSEA) with a false discovery rate (FDR) cut-off of approximately 10%. Nominal P values are also shown, as well as gene set sizes (number of genes in each gene set). No gene sets were significantly down-regulated 7 days after the end of the medication period, with a FDR lower than about 10%.

**Table 4:** Gene sets significantly up-regulated or down-regulated 28 days after the end of the medication period (day 35).

**Rank**	**Gene Sets – Up-regulated**	**Size**	**Nom P-value**	**FDR (%)**
	**Up-regulated**			
1	Protein dimerization activity	60	0.0	3.72
2	Second-messenger-mediated signaling	20	0.0	1.86
3	Endonuclease activity	21	0.0	3.31
4	Protein heterodimerization activity	29	0.0	4.75
5	mRNA catabolic process	20	0.0	4.74
6	mRNA catabolic process, nonsense-mediated decay	17	0.0	6.18
7	Endonuclease activity, active with either ribo- or deoxyribonucleic			
	acids and producing 5'-phosphomonoesters	19	0.0	9.22
8	Endoribonuclease activity, producing 5'-phosphomonoesters	19	0.0	8.07
9	Phosphoprotein phosphatase activity	22	0.0	7.2
				
	**Down-regulated**			
1	Nucleobase, nucleoside, nucleotide kinase activity	25	0.0	0.09
2	Telomerase holoenzyme complex	24	0.0	0.66
3	Prostaglandin biosynthetic process	18	0.0	2.04
4	Prostanoid biosynthetic process	18	0.0	1.53
5	Kinase activity	41	0.0	1.33
6	Nucleotide kinase activity	12	0.0	1.63
7	Kinase regulator activity	11	0.0	2.65
8	Pigment granule	15	0.0	6.8
9	Melanosome	15	0.0	6.04
10	Peptide antigen transport	10	0.0	9.18

Nominal P values are shown, as well as gene set sizes (number of genes in each gene set). Gene set enrichment analysis (GSEA) with a false discovery rate (FDR) cut-off of approximately 10%.

### qPCR verification

**Figure 4:**
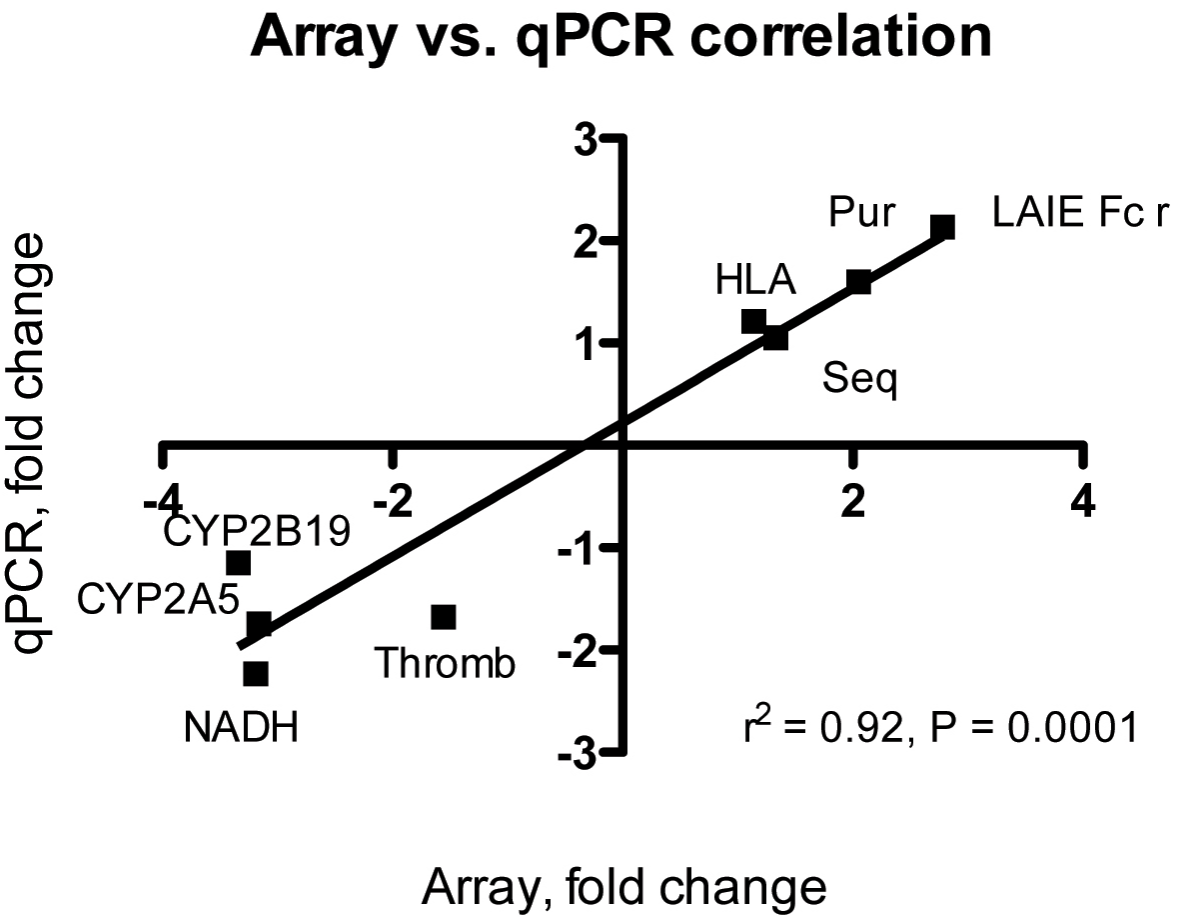
qPCR verification. Linear correlation between microarray and qPCR fold changes. Pur: puridine nucleoside phosphorylase, HLA: HLA class II histocompatibility antogen, gamma chain, Seq: sequestosome, Thromb: thrombospondin 4 precursor, LAIE Fc r: low-affinity immunoglobulin epsilon Fc receptor, NADH: NADH ubiquinone oxireductase chain 1, CYP2A5: cytochrome P450 2A5, CYP2B19; cytochrome P450 2B19.

The qPCR results supported the microarray data (Table 1), with differences only in the scale of estimated up-regulation or down-regulation. Even with the small fold changes in transcriptional levels observed in this experiment there was a significant correlation between the microarray and the qPCR data (Pearson correlation, r^2 ^= 0.92, P < 0.0001; Fig. 4).

**Figure 5:**
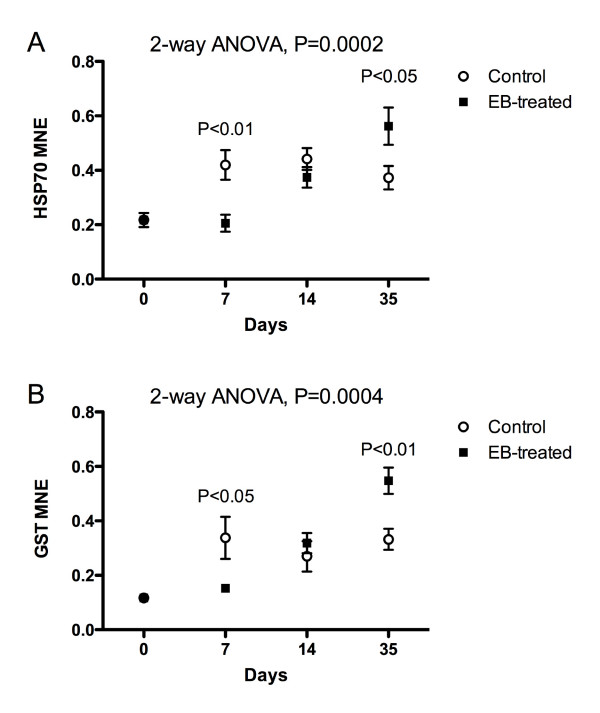
Mean normalized expression (MNE) of A) HSP70 and B) GST in liver of Atlantic salmon (*Salmo salar L*.) orally medicated with the anti-salmon louse medicine SLICE (that contains 50% EB) for 7 days (mean ± SEM.). Samples were taken at day 0 (start of administration), day 7 (end of oral administration), day 14 and day 35. Analyzed by 2-way ANOVA. Overall ANOVA P-value shown in each graph. Significant differences at each time-point are also shown in the graphs. n = 10 in all groups except control day 35 where n = 7.

Independent of the microarray data, the transcriptional levels of two genes encoding stress-responsive proteins were quantified. HSP70 and GST are both well known to respond to external stress in animals, and have often been used as biomarkers. At day 7, the transcriptional levels of both HSP70 (2-way ANOVA, P < 0.01) and GST (2-way ANOVA, P < 0.05) were higher in the control group compared to the EB-treated group (Fig. 5A and 5B). No significant differences were seen at day 14, whereas both genes showed significantly higher mRNA levels at 35 day in the EB-treated groups compared to the control groups (2-way ANOVA; HSP70, P < 0.05, GST, P < 0.01).

## Discussion

Very few genes were significantly differentially regulated in liver (the major detoxifying organ) of EB-medicated fish compared to the control fish at the end of the medication period (day 7), seven days after the end of medication (day 14) and 28 days after the end of the medication (day 35), analyzed by a two class unpaired SAM analysis. Only small fold-change alterations were found in liver, less than two for most of the genes. The results suggest that a standard seven-day treatment with orally administered EB during sea lice treatment (50 μg/kg fish) has only modest physiological impacts on Atlantic salmon.

According to EU legislation the Maximum residue limit (MRL) for EB in fish products for human consumption is 100 ng/g. In all examined tissues the highest EB levels were measured at day 14, seven days after the end of the treatment period. Even at this time point, the EB levels in muscle were below the MRL, suggesting that filet, with the skin removed, is within accepted limits throughout the medication period. The liver in particular, accumulated much higher amounts of EB; at day 14 the level was 9002 ng/g, whereas the concentration in skin was 369 ng/g. These concentrations are in line with earlier published results from examinations of EB-treated Atlantic salmon [9,12]. In a distribution study, Sevatdal et al. [12] reported the highest concentrations in excretory organs, liver and kidney, using autoradiography. High activity in bile suggested that this is the major excretory route. In contrast to our results, they found the highest quantity in liver at Day 7, the last day of administration, whereas in kidney the highest quantities were seen at day 28. Their experiment was performed at higher ambient water temperatures (15–19°C) as compared to the temperature applied in our study (9°C). This may explain the slower distribution of EB to the liver in our experiment. Our results clearly show that EB is easily accumulated in liver of Atlantic salmon and that residues remain in this organ for many weeks. Potential transcriptional effects of EB administration in the fish should therefore most likely be seen in the liver at the end of the medication period or seven days after the end of the treatment.

Gene set enrichment analysis (GSEA) is an analysis method that evaluates the expression of biological pathways on a priori defined gene sets, e.g. biological pathways, rather than looking at individual genes, to identify significant biological changes in microarray data sets [18]. GSEA is therefore especially useful when the transcriptional changes in a given microarray data set are minimal or moderate. GSEA has been applied widely as a tool for gene-set analysis using Gene Ontology (GO) terms. Atlantic salmon is currently not covered by the Gene Ontology Consortium. However GO information is included in the cGrasp annotation file, which contains annotations as of August 2007.

At the end of the medication period (Day 7), nine gene sets were found to be up-regulated (FDR level of 10%) in the treatment group compared to the control group. Six of these gene sets consist of genes encoding proteins involved in protein folding, i.e. isomerase and oxidoreductase activities, partly overlapping between the various gene sets. These proteins can also act as chaperones. Protein disulfide isomerase activity (molecular function: GO:0003756) ranked number one. Among the proteins ranked in these seven gene sets are protein disulfide isomerase precursors (PDI) A2 (PDIA2), A3 (PDIA3) and A6 (PDIA6), sequestosome-1 (Sqstm-1), PDI prolyl 4-hydroxylase subunit beta (P4HB), arginyl-tRNA synthetase (RARS), prostaglandin E synthase 3 (PTGES3), phosphoglycerate mutase 1 (PGAM1), ADP-ribosylation factor GTPase activating protein 3 (ARFGAP3) and arfaptin-1 (ARFIP1). Also included in the top gene set is CYP1A, the major phase I enzyme in the cytochrome P450 system, an enzyme regulated by a number of physiological conditions and xenobiotics. The CYP system is a central catalyst of oxidative reactions including hydroxylation, epoxidation and dealkylation. Although the constitutive level of CYP1A in fish is low, several factors, exogenous as well as endogenous, can affect the CYP1A expression in fish [25]. The major phase I metabolic pathway of ivermectin and EB in rainbow trout and Atlantic salmon is demethylation [9,26]. This may explain the induction of the CYP1A system found in this study. Protein disulfide isomerases are endoplasmic reticulum (ER) resident proteins that catalyze the formation, reduction, and isomerization of disulfide bonds in proteins and are thought to play a role in folding of disulfide-bonded proteins [27]. The majority of disulfide-linked cytosolic proteins are thought to be enzymes that transiently form disulfide bonds while catalyzing redox processes. Cumming et al. [28] recently showed that reactive oxygen species (ROS) can act as signaling molecules by promoting the formation of disulfide bonds within or between redox-sensitive proteins. Our results suggest that EB treatment may have mediated oxidative stress in the liver, a finding, however, not supported by the levels of HSP70 mRNA at Day 7 (quantified by qPCR, Fig. 5A). On the contrary, HSP70 mRNA expression was significantly higher in the control group at day 7, indicating that the control fish might have been stressed during the first 7 days of the experiment. Only at day 35 was the HSP70 mRNA level significantly higher in the medicated group. Heat shock proteins are a family of highly conserved proteins that protect the cells against cytotoxic effects of protein degradation [29]. Many types of external stress can induce HSPs in fish, including oxidative stress [30,31], even though their value as biomarkers of various form of stress in fish has been questioned [32]. Judged by the enriched gene sets at day 35, oxidative stress seems to have been a transient response, since none of these gene sets contained HSPs.

Neither GST π showed increased transcriptional levels at day 7 (Fig. 5B); only at day 35 was the transcriptional level of GST significantly higher in the EB-treated group compared to the control group. The GSTs are a family of biotransforming enzymes that protect cells against injury from a number of endogenous and environmental chemicals. Trute et al. [33] characterized GSTs in Coho salmon (*Oncorhynchus kisutch*). They found two major GST isoforms in liver, the π and θ-class GSTs, but noted that they might have a limited capacity to conjugate substrates of various toxicants and endogenous compounds associated with cellular oxidative stress. We have recently shown that the GST π class is inducible by β-naphthoflavone in Atlantic salmon [34]. The results presented here suggest that that the π class GST is inducible by EB in Atlantic salmon and that EB may undergo glutathione conjugation. This metabolite has however not yet been described in fish.

The other three gene sets up-regulated at Day 7 in the EB-medicated fish consist of genes encoding proteins involved in protein modification processes (GO:0006464), i.e. peptidyl-asparagine modification [GO:0018196]. Proteins encoded by genes in these gene sets include among others dolichyl-diphosphooligosaccharide-protein glycosyltransferase (defender against cell death 1, DAD1) and various DAD1 subunits, keratinocyte-associated protein 2 (KRTCAP2), strumpellin (KIAA0196), alpha-1,6-mannosyl-glycoprotein 2-beta-N-acetylglucosaminyltransferase (Mgat2), arginine/serine-rich coiled coil protein 1 (RSRC1) and cytochrome c oxidase subunit-1 (MT-CO1). DAD1 was initially identified as a negative regulator of apoptosis in the BHK21-derived tsBN7 cell line, and is a subunit of the mammalian oligosaccharyltransferase [35]. It has been shown that loss of the DAD1 protein triggers apoptosis [35]. The thrombospondin-4 precursor (THBS4), belonging to the thrombospondin family, a group of proteins involved in the positive regulation of apoptosis, was significantly down-regulated at day 7, verified by qPCR analysis. These findings suggest that EB treatment may have affected the regulation of apoptosis. Although involved in a number of biological processes, the proteins encoded by the genes comprising these two pathways, suggest that EB treatment might have affected protein stability and folding at day 7, possible via induced oxidative stress.

The most significant up-regulated gene sets at day 14 suggest that EB treatment induced an inflammatory response (prostaglandin biosynthetic process GO:0001516) in the liver. Proteins encoded by genes in the top six gene sets include prostaglandin E synthase 3 (PTGES3), HLA class II histocompatibility antigen gamma chain (CD74), H-2 class II histocompatibility antigen gamma chain (Cd74), H-2 class II histocompatibility antigen, A-B alpha chain precursor (H2-Aa), fibrinogen gamma chain precursor (Fqq), myosin-9 (Myh9), hematopoietic SH2 domain-containing protein (Hsh2d), zinc finger protein 706 (ZFP706) and 15-hydroxyprostaglandin dehydrogenase (NAD^+^) (Hpgd). Prostanoids, including eicosanoids and metabolites of eicosapolyenoic fatty acids, are not stored by cells but rather synthesized in many cell types in response to cell-specific proteolytic or hormonal stimuli [36]. These processes are usually receptor-mediated, but may also be elicited by mechanical stresses on cells [36]. Up-regulation of prostanoids further suggests that EB treatment mediated oxidative stress, as free radicals may oxidize unsaturated fatty acids [37], i.e. eicosanoids. Surprisingly, the prostanoid pathways were down-regulated at day 35, suggesting a counter-reaction in gene transcription.

Several gene sets were differentially changed at day 35, indicating that EB still affected transcription in hepatic cells one month after the end of the medication period. Protein dimerization activity (GO:0046983) was listed as the most up-regulated gene set. Also up-regulated was endonuclease activity (GO:0048256). These findings suggest that EB treatment affected protein binding and nucleotide cleavage even at day 35, although further examinations are needed in order to elucidate to long-term effects of EB medication in salmon.

qPCR is a commonly used validation tool for microarray analysis. Microarray and qPCR data often disagree, and no standard definition for validation exists [38]. It is well documented that both qPCR and microarray analysis have inherent pitfalls that may influence transcriptional levels quantified with each method. In this work we picked 8 of the most significant genes from the SAM analysis for qPCR verification, of which most were changed less than 2-fold. The qPCR data were in line with the array data, although the down-regulated genes showed higher discrepancy than the up-regulated genes, for an unknown reason.

## Conclusion

In conclusion, this study has shown that a standard seven-day EB treatment has only modest effects on the transcription of genes in liver of Atlantic salmon. Based on GSEA, the medication seems to have produced a temporary oxidative stress response that appeared to affect protein stability and folding, followed by a secondary inflammation.

## Authors' contributions

PAO and BTL planned and designed the experiment. PAO carried out the transcriptional analysis and drafted the manuscript. KKL and EM were involved in qPCR and microarray work. BTL and OBS made the pharmacokinetic study. KP and AKS were involved in experimental design and statistical analysis of the microarray experiments. All authors read and approved the final manuscript.
